# Metropolitan Hospital

**Published:** 1906-07-07

**Authors:** 


### METROPOLITAN HOSPITAL.

The Metropolitan Hospital held its festival dinner at the Whitehall Rooms on Monday, the Hon. Robert Guinness presiding. Amongst those present were Lord Howard de Walden, the Hon. Claude Hay, M.P., and Sir E. Hay-Currie. During the year 1905 the in-patients increased by 121, from 1,220 in 1904 to 1,341, and the out-patients by 2,545, from 36,167 to 38,712. The attendances of the latter amounted to 128,088, from which it is evident that steps will have in the near future to be taken to enlarge this department of the hospital. The average stay of each in-patient was reduced from 28.66 in 1904 to 27.98 in 1905. Although the ordinary receipts had increased from £11,229 to £11,420, the total income was £1,938 less than in 1904, owing to legacies only amounting to £2,377 against £4,508 in 1904. The ordinary expenditure was slightly reduced, being £14,174 (£276 less than that of the previous year), and the average cost of each in-patient was brought down from £2 2s. 10d. in 1904 to £1 18s. 3d. in 1905.

The President, in proposing "Prosperity to the Hospital," said that the experience he had gained as a Parliamentary candidate in the neighbourhood of the Metropolitan Hospital had taught him that the inhabitants of the district in which the Metropolitan Hospital was situated ran a very poor chance of receiving adequate nursing in their own homes, therefore the necessity for such a hospital in their midst. The inhabitants of that district were not people who did not try to help themselves. They did try, and one of the means by which they endeavoured to do so was by the formation of hospital societies, the members of which subscribed a penny a week, out of which were obtained hospital letters for distribution amongst the members.

Lord Howard de Walden, the Chairman of the Hospital, in responding, divided hospitals into three classes—the blatantly prosperous hospitals, the imposing but extremely shaky hospitals, and the unconsidered hospitals. The Metropolitan Hospital was in the last mentioned category.

The Secretary announced that the result of the collection on behalf of the hospital amounted to £4,487.

Sir E. Hay-Currie proposed "The Committee of Management." He said that though the result of the Hospital Sunday collection was not yet known, he did not think it would be so good this year as usual; the rich congregations of London had not of late years sent in such large contributions as they had done in the past. The hospitals of the metropolis were not being supported as they should be. There were to his knowledge at least two of the large London hospitals which were literally living from hand to mouth and from day to day. If that was so with two hospitals placed so that they were seen by the millions of London day by day, what must be the case of a hospital like the Metropolitan, situated out of sight in the Kingsland Road. Fortunately, however, the hospital had an excellent Committee of Management, a capital Chairman in Mr. C. J. Thomas, who was well backed up by his committeemen, and they had also this tremendous advantage that they had one of the best secretaries in London in Mr. Charles Byers. (Loud Applause).

Other toasts followed.

